# Absolute abundance of southern bluefin tuna estimated by close-kin mark-recapture

**DOI:** 10.1038/ncomms13162

**Published:** 2016-11-14

**Authors:** Mark V. Bravington, Peter M. Grewe, Campbell R. Davies

**Affiliations:** 1 CSIRO, Castray Esplanade, Hobart, Tasmania 7000, Australia

## Abstract

Southern bluefin tuna is a highly valuable, severely depleted species, whose abundance and productivity have been difficult to assess with conventional fishery data. Here we use large-scale genotyping to look for parent–offspring pairs among 14,000 tissue samples of juvenile and adult tuna collected from the fisheries, finding 45 pairs in total. Using a modified mark-recapture framework where ‘recaptures' are kin rather than individuals, we can estimate adult abundance and other demographic parameters such as survival, without needing to use contentious fishery catch or effort data. Our abundance estimates are substantially higher and more precise than previously thought, indicating a somewhat less-depleted and more productive stock. More broadly, this technique of ‘close-kin mark-recapture' has widespread utility in fisheries and wildlife conservation. It estimates a key parameter for management—the absolute abundance of adults—while avoiding the expense of independent surveys or tag-release programmes, and the interpretational problems of fishery catch rates.

## Abstract (web summary)

Accurate determination of population size for highly-mobile marine animals is often prohibitively difficult. Here, Bravington *et al.* estimate the abundance of southern bluefin tuna using a method based on the number of parent-offspring pairs detected genetically in samples from the catch.

Abundance estimation is a fundamental challenge in ecology and the management of exploited populations. Nowhere is this truer than in the oceans, where animals may be widely distributed, highly mobile and difficult to observe directly. Here we present a new way to estimate abundance—and other key parameters such as mortality rates—that only requires small pieces of tissue, taken from either live animals or dead ones (for example, commercial fish catches). Close-kin mark-recapture (CKMR) makes use of advances in genetics to affordably and reliably identify parent–offspring pairs (POPs; and conceivably other types of kin), and then analyses the number and pattern of pairs in a mark-recapture framework. We present a successful application to southern bluefin tuna (SBT; *Thunnus maccoyii*), a highly migratory, heavily depleted species that supports a valuable international fishery (>$1bn p.a.), yet where traditional but highly contentious data sources have left great uncertainty about its status and recovery prospects. CKMR bypasses the dependence on problematic fishery-derived data, while avoiding the expense of fishery-independent studies such as dedicated surveys or tagging programmes. It promises to revolutionize the monitoring of previously intractable species in marine, freshwater and terrestrial environments.

The three bluefin tuna species exemplify the difficulties of traditional abundance estimation for marine fish^1^,^2^,^3^,^4^. Most populations of these highly valued, wide-ranging animals have been subject to overfishing by multiple international fleets over decades, resulting in depleted populations and significant economic loss^5^,^6^,^7^,^8^. A major challenge to reaching international consensus on management action has been uncertainty about the absolute abundance of each stock, and the level of depletion of its reproductive component^6^,^9^. As in most fisheries, the primary abundance index for bluefin tuna stock assessments is standardized catch per unit effort (CPUE) derived from the fishery itself^10^. The hope is that true changes in abundance are reflected by proportional changes in standardized CPUE, but in practice the presumed relationship can be compromised by ‘unstandardizable' changes in fishing practice and spatial coverage, as well as by misreporting. Thus, different interpretations of changes in CPUE have been a major source of contention in bluefin tuna^11^,^12^,^13^ and in fisheries generally^14^,^15^. For certain fish species, directed scientific surveys and/or individual mark-recapture can provide fishery-independent alternatives to CPUE; however, these alternatives tend to be costly and may still come with interpretational problems of their own (for example, tag return rate uncertainty in mark-recapture^16^, even when technically feasible). To date, there simply has been no realistic fishery-independent alternative to CPUE for the main targeted age classes of bluefin tuna.

Our alternative CKMR approach rests on two simple ideas. First, modern genetics allows us to tell reliably whether any two fish constitute a POP; second, all juveniles have two parents. The principle is shown in the hypothetical and simplified Fig. 1. In effect, each juvenile genetically ‘marks' or ‘tags' its two parents among a total adult population of size *N*_adult_, which is presumably large but unknown and to be estimated. Assume for now that maternity/paternity testing is completely accurate. When a sampled juvenile is compared genetically with a randomly sampled adult, the chance that the pair will turn out to be a POP—i.e, that the adult will have one of the two marks from the juvenile—is 2/*N*_adult_. To estimate *N*_adult_, we could therefore genotype a number of adults and juveniles (*m*_A_ and *m*_J_), conduct all pairwise comparisons, and count how many POPs are actually found (*P*). The expected total of POPs is the number of comparison times the probability that each one will yield a POP, that is, *m*_J_*m*_A_ × 2/*N*_adult_. Therefore, the observed total yields a natural estimate of adult abundance that is directly analogous to a Lincoln–Petersen abundance estimate in standard mark-release recapture:


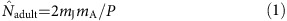


It is not necessary to assume that all adults make an equal reproductive contribution; even if some adults have more offspring than others, as in Fig. 1b, the number of marks (lines) is the same as in Fig. 1a, although the sampling variability of the estimate is clearly greater in Fig. 1b. There is no bias in the abundance estimate, provided that the chance of an adult being sampled is independent of how many offspring it happens to have in the sample. This condition can be relaxed in real applications, as described below.

Close-kin pairs (including but not limited to POPs) have been used before in demography, particularly for connectivity^17^,^18^. CKMR-like frameworks for abundance estimation were first proposed in 2001 (refs 19, 20); however, the scope was limited by the technical and financial difficulties in genotyping thoroughly enough to distinguish known degrees of kinship, especially among the large samples required for large populations. This led to modelling and/or sampling complications, for example, where the trend and abundance are statistically confounded^20^, or where mothers, calves and fathers need to be genotyped^19^. In this paper, we take advantage of the improved reliability and affordability of genotyping to focus purely on POPs and to keep a small, calculable margin of error; our approach leads to a simpler but more extensible mark-recapture framework, as well as to a sampling programme that is practicable even for large populations (many millions of adults). The Methods section provides more detail on the genotyping procedures we followed; CKMR demands exceptionally tight control of error rates to avoid a small number of true POPs becoming swamped by false-positives.

In more realistic settings than Fig. 1, an explicit statistical mark-recapture model is needed, in effect adjusting the term 2/*N* for each pairwise comparison to take account of pair-specific covariate data such as sex, year and age. The prior probability that a pair is a POP is set by a population dynamics model, which accounts for pair-specific data, and which includes demographic parameters such as adult abundance, mortality (because the parent might have died between juvenile birth and adult capture) and perhaps age-specific fecundities. Similar models are the basis of any structured fish stock assessment; however, here the model is used to compute probabilities of POPs rather than, say, expected catch rates. For example, when checking whether individual *i* with covariates *z*_*i*_ (date of capture for example) is the mother of individual *j* with covariates *z*_*j*_, the formula would typically be


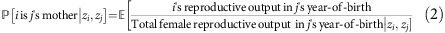


where 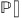 is probability, 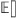 is expected value and the absolute abundance (of females) now enters implicitly through the denominator. Some care is needed to implement equation (2) appropriately and avoid bias, especially if some important covariates are unobserved (for example, age). As with the cartoon in Fig. 1, the key requirement is that *i*'s chance of being sampled is independent of how many of her offspring happen to be sampled; however, now independence need only apply conditional on the covariates. This framework allows, for example, models where bigger adults can be more catchable as well as more fecund (Supplementary Note 1). Demographic parameters can then be estimated from the log-likelihood summed over all pairwise comparisons, either by maximum likelihood or by Bayesian methods^21^.

In Methods, we describe a length-, sex- and age-structured population dynamics model for adult SBT, which we used to combine close-kin comparisons, time series of length- and age composition of the adult catches (fishery-derived, but non-contentious), and biological studies of growth and daily fecundity. From 38,000,000 pairwise comparisons, which yield 45 POPs in total (error bounds ±1), the resulting abundance estimate is considerably higher than previously suggested by traditional stock assessments, which had to rely on untestable assumptions about major gaps in historical data. CKMR also provides direct evidence on which animals are contributing surviving offspring, and for SBT it turns out that the reproductive contribution of big females must be disproportionally greater than bodyweight alone suggests—again in contrast to previous untestable assumptions, and with important implications for the productivity and resilience of the species.

## Results

### SBT and CKMR

SBT is a large (2+ m), long-lived (40+ years), late-maturing (age 8+) fish^22^,^23^. It ranges across the four oceans of the southern hemisphere, with a single spawning ground south of Indonesia in austral summer, and juvenile summer feeding grounds in the Great Australian Bight^3^,^24^,^25^,^26^. SBT has been harvested by coastal and international fleets since the early 1950s, leaving the spawning stock depleted to below 10% of estimated pre-exploitation levels^27^, although the exact level is uncertain. The main current fisheries target juveniles (ages 1–4) by purse-seine in the Great Australian Bight, and subadults (ages 3–10) by longline on the high seas and coastal waters of Australia, New Zealand and South Africa. Smaller numbers of adults are also caught on the spawning grounds (and invariably in spawning condition) as bycatch of a tropical tuna fishery^22^. The fisheries are managed internationally through the Commission for the Conservation of SBT (here ‘the Commission').

As with most tuna stocks, the SBT stock assessment relies on CPUE from longlining as a primary index of (relative) abundance. This index suffers from all the complications that plague CPUE in other fisheries: incomplete coverage of the population range; time-varying spatial dynamics of the fish and fleets; lack of independent verification of catch and effort; restrictive data access that limits transparency of assessment inputs. In addition, revelations of large, long-term unreported catches through to the mid-2000s further undermined confidence both in the interpretability of CPUE as a relative abundance series^28^,^29^, and in the calibration to recent absolute abundance that requires absolute catches. The Commission's Scientific Committee was thus forced to construct ‘scenario analyses' about historical catch and effort, in lieu of a traditional stock assessment^30^. While just about any marine fishery would benefit greatly from an absolute estimate of adult abundance that does not depend on CPUE nor on direct surveys nor on tagging, for SBT in particular the need is acute.

To derive instead a close-kin-based abundance estimate for SBT, we collected over the period 2006–2010 tissue samples from 5,755 adults caught on the spawning grounds in the spawning season, and from 7,448 3-year-old juveniles caught in the Great Australian Bight during the austral summer. Length and sex were recorded for all adults, and age (from otoliths) for about one-third. We developed a suite of highly variable but reliably scorable microsatellite loci, and genotyped each sample at up to 25 loci; overall, 92% of loci were successfully genotyped, and 94% of fish were genotyped at ≥80% of loci attempted. All pairs of adults and juveniles were compared, provided that the adult was caught after the juvenile birth year, and that the *a priori* false-positive probability for the pair was not too high based on the number of loci scored in both members (thus eliminating ∼7% of potential comparisons). Among roughly 38 million pairwise comparisons, we found 45 POPs, accurate to ±1 in terms of false-positives and false-negatives (Supplementary Methods 1). To estimate abundance and other population parameters, we developed a length-, sex- and age-structured population dynamics and mark-recapture model for the pairwise POP comparisons and for the adult length/age/sex compositions (Methods). The main conclusions from fitting this model are as follows.

### Sibship

Each of the 45 POPs involves a distinct adult and a distinct juvenile (that is, no fish occurs in more than one POP). This is consistent with a low proportion of siblings among our 3-year-old juvenile samples (something supported by other genetic analyses, in Supplementary Methods 1), which is the main requirement for an unbiassed coefficient of variation (CV; see Supplementary Note 3).

### Size-specific fecundity

**Figure 2:**
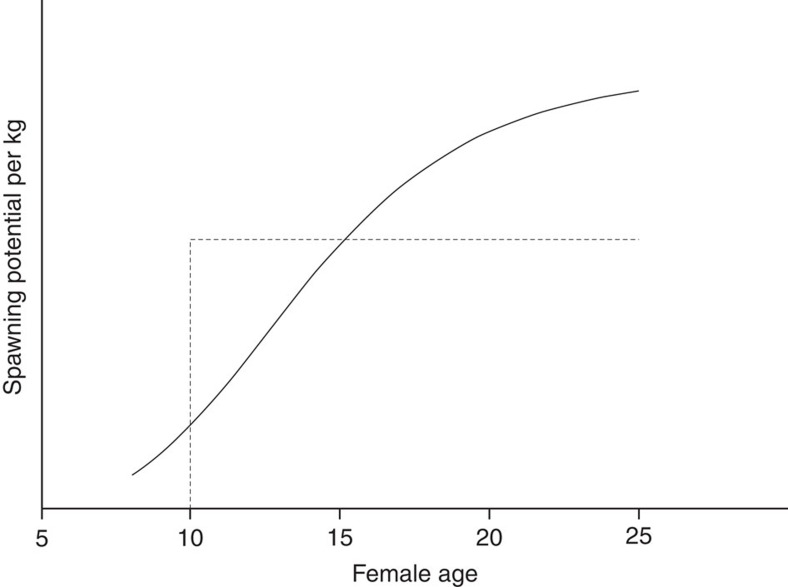
Reproductive output per kg of female bodyweight as a function of age. Shown by solid line for average female of that age. Inferred from estimated length-at-age distributions and estimated relative reproductive output by length. Dotted stair-step line corresponds to previous ‘knife-edge' assumption.

Larger, and thus usually older, female SBT contribute reproductively beyond their weight. This can be inferred by comparing the length distributions of identified mothers to that of adult females. Both distributions are affected similarly by (i) selectivity and (ii) the true population length composition. However, the observed length distribution of mothers is also weighted by annual fecundity (measured in terms of surviving 3-year-old juveniles); more-fecund mothers are ‘tagged' more often by their surviving offspring. In a multi-year study, direct comparison of distributions is not meaningful because of adult growth and consequent selectivity changes between juvenile birth and adult ‘recapture'; therefore, it is necessary to fit the full population dynamics model. With just 20 of the 45 parents being female, precision is limited, but the overall effect is significant (*P*<0.01, Wald test) and the estimated relationship is strongly nonlinear in bodyweight (Fig. 2); an average 15-year-old female contributes ∼3.2 times as many surviving offspring per kilo as an average 10-year-old, and an average 25-year-old ∼1.4 times as much again. This is contrary to the assumption—previously used in SBT stock assessments, widespread in fisheries, and generally untestable—that reproductive contribution is proportional to bodyweight after maturity. In addition, maturity for SBT has traditionally been assumed to occur suddenly at the age of 10, but the two youngest identified male and female parents in this study were in fact aged 8 when their offspring were born.

### Adult survival rate

The average annual survival rate of adults is estimated at 0.77 (s.e.=0.025). Adult mortality rates cannot be estimated just from annual age and length compositions because of statistical confounding with fishery selectivity^31^, and the fishing mortality on spawning adult SBT is too low for conventional approaches to work. However, the POPs provide another source of information, intuitively because the average interval between juvenile birth and adult ‘recapture' is lower if the adult mortality rate is higher. By incorporating additional information on female daily fecundity^22^, the adult mortality rate for SBT becomes statistically estimable.

### Abundance

**Figure 3:**
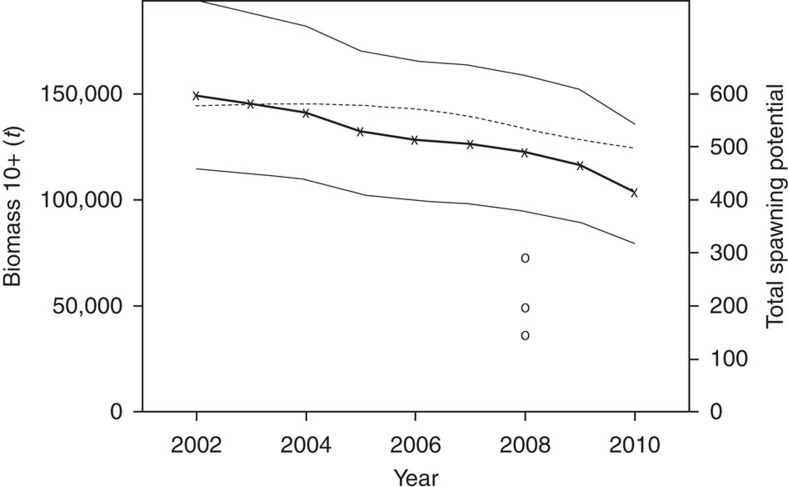
Estimated trends in adult biomass and spawning potential. Time series of adult biomass estimated by CKMR (‘adult' taken here to be age 10+ to match previous usage in stock assessments), shown as the dark solid line with crosses; 90% confidence intervals are shown as narrow solid lines. Comparable point estimates of adult biomass in three scenarios from a pre-CKMR stock assessment are shown by circles. The dashed line shows total female spawning potential as estimated by CKMR (in units of ‘equivalent 1,000 age-16 females').

The CKMR absolute abundance estimates are fairly precise; for example, the CV of adult biomass in 2008 (ages 10+) is ∼0.17, not much higher than the theoretical minimum of 0.15 imposed by observing just 45 POPs. Information on trend is less certain. The estimated time series of adult biomass is shown in (Fig. 3), along with the time series of total spawning potential, integrated over all female adults (here taken to be aged 8+) and allowing for the effect of body size (and therefore age) on fecundity. The two trends are appreciably different because of the new fecundity-at-size estimates combined with changing adult age composition. For comparison, also shown are the point estimates from three scenarios considered in the Commission's 2011 stock assessment, which was the last assessment before the advent of CKMR data (these scenarios were then referred to as ‘optimistic', ‘base case' and ‘pessimistic', primarily based on their assumptions about historical catch and effort—data not used by CKMR). The pre-CKMR point estimates are widely spread and are two to three times lower than the CKMR estimates.

Subsequently, the POP data and CKMR likelihood have been integrated directly into the Commission's stock assessment, along with other fishery data. Despite the apparent large difference in point estimates of abundance (Fig. 3), the CKMR data and other data do turn out to be compatible once the stock assessment has been adjusted to take account of qualitative (structural) insights from CKMR, in particular the fecundity-at-size result above^32^,^33^,^34^. The CKMR data have substantially reduced uncertainty about adult abundance and depletion, ruling out the pre-CKMR 2011 stock assessment scenarios with lowest assumed biological productivity (for example, corresponding to the lowest circle in Fig. 3). The estimated ratio of spawning stock in 2011 relative to pre-exploitation levels improved from 5% without close-kin data to 8% with it: still heavily depleted, but appreciably less so than formerly thought, and with substantially better prospects for stock-rebuilding.

Recognizing the value of CKMR as a cheap and direct measure of adult abundance, the Commission has funded continued sample collection and design studies for long-term monitoring to independently assess progress against the stock-rebuilding plan^32^,^35^.

## Discussion

We have shown that absolute abundance, and other demographic parameters important for management, can be estimated by ‘mark-recapture without marking'. Samples can be collected just from the catch (or other sources of anthropogenic mortality), and then modern genetic techniques can identify POPs, which become the ‘recaptures' in a mark-recapture model for parameter estimation; the ‘marks' are an automatic genetic consequence of inheritance. CKMR offers a breakthrough for many commercial fisheries, and other ecological applications without a cost-effective monitoring tool because of compelling advantages over more traditional data sources: low cost, logistic simplicity, few assumptions and lack of susceptibility to reporting biases.

The CKMR approach is quite general, even though our application to SBT has some unusual features that might at the first glance seem limiting. For example, adult and juvenile SBT are physically separated and caught in different fisheries; while this does simplify explanation, there is no reason why equation (2) cannot be applied to the more common case where fishing is mainly on adults, and adult–adult comparisons are used instead. If live release is possible, CKMR could of course be conducted with biopsies too, and could be combined with individual mark-recapture. Spatial population structure, which is an issue for many species although not SBT, conceptually requires rewriting equation (2) to integrate over possible birthplaces. However, since CKMR data are informative in their own right about population structure based on the proximity within identified kin pairs^17^,^36^, the mere existence of population structure may not be limiting.

Population size also affects logistics. The required sample size to achieve some target precision is set by the need to find a reasonable number of kin pairs, and scales with 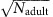 (Supplementary Notes 2 and 3). Thus, while bigger populations do need bigger sample sizes in absolute terms, the sample size relative to abundance (and thus to potential catch) is actually lower. For SBT, we estimate that the cost of this project was under 0.1% of the value of the global fishery over the 5-year duration: very good value, given the quality of information acquired and the absence of other methods of getting it. Even for typical groundfish species, say with abundance maybe three orders of magnitude higher and with bodyweight (and unit value) an order of magnitude smaller than SBT, the economic proposition may still be compelling. Longer studies aimed at time series are even better value (Supplementary Note 2). There are limits: profoundly abundant species like krill are unlikely ever to be affordable, and certain life-history traits (for example, semelparity and parthenogenesis) make CKMR inapplicable. That aside, the main limit is likely to be whether samples (and ancillary data such as age) can be obtained from relevant life stages.

Genetic technology has improved rapidly over the last decade, and will continue to do so. In 2005 when we began planning our study, microsatellites were the best proven option; however, for a study starting now single-nucleotide polymorphisms (SNPs) would likely be preferred (either sequencing-based genotyping^37^ or targeted assay^38^). As of 2016, unit costs are roughly comparable across technologies; however, the one-off cost of locus development, which was high for our carefully chosen microsatellites, is now much less for SNPs owing to sequencing-based methods. This will make CKMR much more affordable for species of low economic value.

Parent–offspring CKMR should no longer be thought daunting from a genetic perspective, but it does require careful design both of sampling and of genetics, large sample sizes (much larger than, say, for population genetics), commensurate budgets and the ability to develop bespoke statistical mark-recapture and population-dynamic models. In the near future, we expect to see two main uses for CKMR: commercial fisheries, particularly for species of high individual value and high abundance, where sample sizes are large but the cost-to-value ratio is especially compelling; and threatened species (terrestrial as well as marine) where the required sample size and the absolute cost of genotyping is low, and concern is enough to justify the effort.

## Methods

### Sampling and biological data collection

Adult muscle tissue samples were collected from Indonesian SBT longline landings throughout the 2006–2010 Indonesian fishing seasons (October–March), using trained samplers in an ongoing catch-monitoring programme in Benoa, Bali^39^,^40^; over 90% of the Indonesian SBT catch is landed there and at least 25% of the landed SBT were monitored each year. This fishery, which is mainly aimed at other tuna species, covers most of the only spawning area of SBT. Length and sex were recorded for all monitored fish, and age was estimated from otoliths for a length-stratified subsample of 500 fish per year, as well as subsequently for all identified parents^41^. All tissue samples were frozen to −20 °C and then shipped to Australia for genotyping. About 1,300 adults were genotyped annually from 2007 onwards (total 5,755).

For juveniles, muscle tissue samples were taken from 4,000 fish per year between 2006 and 2010 at point-of-harvest in Port Lincoln, South Australia (where wild juveniles are caught offshore, and then grown on in pens for several months before harvest). The samples comprise 2-, 3- and 4-year-old fishes, with well-separated modes in the length frequency distribution^42^. For genotyping, we chose only fish with lengths close to the mode for 3-year olds, genotyping ∼1,500 of them annually between 2006 and 2010, along with ∼200 4-year olds from 2006 (total 7,488).

### Genetic identification of POPs

There are several methods for establishing parentage^43^,^44^, all based on the same principle of Mendelian inheritance. A true POP must have at least one allele in common at each locus because of inheritance. An unrelated pair may also share an allele by chance at some loci, but if enough loci are examined then it becomes very unlikely that all loci in an unrelated pair will happen to contain a shared allele (that is, that no examined locus will exclude the possibility of parentage). Consequently, the exclusion principle, sometimes referred to as the ‘gold standard for parentage'^44^, accepts the pair as a POP if and only if every locus contains a shared allele. It is always possible to get a false-positive POP by chance, but by using enough loci the probability can be kept below any desired target. This per-comparison false-positive probability may need to be very low for CKMR, where for reasonable precision only ∼10^2^ true POPs are needed but in a large population ∼10^8^ (in our example) or more comparisons may be needed. The number of loci required to achieve a given false-positive rate can be predicted based on preliminary allele frequency estimates for each locus obtained from genotyping a modest number of samples. For large populations, the number of loci required to rule out false-positive POPs from unrelated pairs will also be enough to rule out false-positives from non-parental kin such as grandparent–grandchild pairs; although non-parental kin pairs have individually higher false-positive probabilities, they are far rarer than unrelated pairs (Supplementary Methods 1).

False-negatives could also arise, whereby a true POP is overlooked because a genotyping error (or, less likely, a mutation) leads to exclusion at one or more loci. This possibility can be handled by genotyping every sample at several more loci than the bare minimum just indicated, enough so that even the ‘luckiest' unrelated pairs would be statistically sure to exclude at, say, three or more loci. Consequently, any pair that seems to have just one parentage-excluding locus is almost surely the result of a true POP with a genotyping error or a mutation. The proportion of such pairs found allows *post hoc* assessment of the false-negative rate. Some modifications of the strict exclusion principle may be required to allow for genotyping errors and null alleles (Methods).

The genetic design issues for parentage-based CKMR therefore amount to (i) choosing the type of loci (microsatellites, as in our study, or SNPs) and the genotyping system, (ii) careful locus screening and protocols to minimize genotyping and POP-detection errors, and (iii) using enough loci to control and bound the likely number of false-positive and false-negative POPs.

### Locus development

The microsatellites already available for SBT in 2006 had been developed for population genetics, where an abundance of rare alleles is statistically undesirable. For efficiently finding close-kin, though, it is desirable to have many rare alleles, because this increases the per-locus chance of parentage exclusion in pairs that are not parent–offspring, so that fewer loci are needed and overall costs are lower. Reliability of scoring is also more critical for parentage than for population genetics. We therefore developed a suite of new microsatellites specifically for SBT parentage that (i) were highly variable, but not so variable that the longest alleles failed to amplify well; (ii) had solitary and sharp peaks, with minimal shoulder to the peaks and little stutter; and (iii) had clear gaps between alleles. We concentrated on tetranucleotide loci, i.e., with allele lengths in steps of four base pairs.

We genotyped the first 5,000 fish at 20 loci organized into five monoplex and multiplex panels. If all loci could be scored successfully in all samples, then those 20 would be adequate to exclude false-positives. However, a modest proportion of early cases did prove to be unscorable, which increases the potential for false-positives overall. Based therefore on interim results about the rate of unscorable loci and of likely final sample size, we developed another five loci and reorganized the panel layout; therefore, the remaining 8,000 fish were scored at 25 loci on four multiplex panels (plus another two loci that were slightly harder to score, and which were used for quality control but not parentage). The false-positive and false-negative bounds were satisfactory (Supplementary Methods 1); therefore, we did not re-genotype the first 5,000 fishes at the new loci.

### Scoring protocols, locus quality control and parentage-exclusion criteria

The FSA files from GeneMapper were scored by one of four experienced people, each scoring several thousand samples. About 10% of the samples were rescored from the same FSA files by another reader for internal quality control. Consistency checks on allele-calling are described in Supplementary Methods 2. The protocol was to record ‘unscored' if in doubt, particularly about the presence of a possible second peak. Most (20 of 25) loci showed a small excess of homozygotes (Supplementary Table 1), interpreted as true null alleles either from heritable mutations or from scoring errors where a peak is overlooked. Allele frequencies were estimated (from at least 8,000 samples per locus) by maximum likelihood allowing for nulls, but ignoring unscored loci (that is, similar to ref. 45, except being conditioned on finding at least one non-null allele). Since heritable nulls could lead to spurious parentage-exclusion (if an A0 parent has a B0 offspring), we modified the parentage-exclusion criterion to tolerate comparisons of apparent homozygotes (AA/BB); in the end, there were nine such cases found among the 45 POPs, at ∼1% of all loci. The computation of all locus-specific false-positive probabilities made allowance for null alleles and used the modified criterion.

We fitted a generalized additive model (GAM) to check for any relationship between overall DNA quality (as measured by proportion of scorable loci for each fish) and reported homozygosity among those loci that were scored. There was no evidence of any relationship, except for fish scored at so few loci that they would in any case be omitted from all pairwise comparisons. There was also no evidence of long-allele dropout (among apparent homozygotes at each locus, no significant relationship between allele length and excess of homozygotes, after accounting for allele frequency). The large sample size (*n*≥8,000 fish scored at 25 loci) gave good power to detect such locus-level problems.

Because most fishes have one or more loci which could not be reliably scored, most pairwise comparisons involve fewer than the maximum 25 or 20 loci. The chance of a false-positive (no parentage-excluding loci found) depends on how many loci are used, and which ones. To ensure that the overall proportion of false-positives is low, we first compute the parentage-exclusion probability of each pairwise comparison based only on which loci were scored (not on what the scores were), and then retain only the most informative comparisons such that the overall expected number of false-positive POPs is under 1% of the actual number of POPs found. We also retain only those comparisons where the adult was caught after the birth year of the juvenile; a POP is clearly impossible if the adult is caught before the juvenile is born, and adults caught part-way through a year have not made their full reproductive contribution, so equation (2) does not apply. While other types of non-POP kin pairs are individually more likely to give a false-positive POP than an unrelated pair is, the total expected number of false-positives from non-POP kin pairs is negligible because they are demographically far rarer than unrelated pairs in a large population.

However careful the protocols are, genotyping errors are still possible, and could lead to false-negatives where a true POP is overlooked because of one or more apparent parentage-excluding loci^46^; note that genotyping errors in unrelated pairs do not increase the probability of false-positives. The possible impact of small-scale genotyping errors (that is, statistically independent errors at the level of individual fish) can be examined by tabulating the numbers of apparently parentage-excluding loci across comparisons. The POPs stand out clearly in the left-hand column of Supplementary Table 2, which shows retained adult-juvenile comparisons; as a sanity check, this column is almost empty in Supplementary Table 3 where juveniles are compared only with other juveniles so that true POPs are impossible. Detailed statistical analysis of Supplementary Table 2 leads to formal statistical bounds on false-negative POPs (95% confidence interval of 1 or less). Further analysis of Supplementary Table 3 also demonstrates that there cannot be a problematically high proportion of within-year siblings (full and/or half) among the juvenile samples (Supplementary Methods 1).

Large-scale genotyping errors would not be detected this way; with a large-sample multipanel study like this, panels may occasionally become swapped and/or rotated, so that the genotypes of an entire group of fish may be mis-recorded as ‘chimeras', each chimeric genotype containing loci from two or more fishes. We used control positions, guard loci and duplicate rows to detect (rarely) and fix such problems (Supplementary Methods 2).

To confirm POPs, we rescored (from the same FSA files) all retained pairs that had no more than two parentage-excluding loci. The rescoring, which was conducted blind (that is, without knowing what the pairings might be), did detect one previously false-negative POP, where a heterozygote had been scored as a homozygote. For a few pairs (for example, the pair at C25,X2 in Supplementary Table 2) we repeated the entire genotyping process, starting with DNA extraction. Subsequent analysis with a SNP panel suggests that this pair is either grandparent–grandoffspring or half-sibling, but certainly not a mis-scored POP.

### Population dynamics

We use a standard single-stock age-structured two-sex population dynamics model for adult SBT, covering years 2002–2010 (earliest birth of genotyped juvenile to latest capture of genotyped adult) and from age 8 (the youngest successful breeding age of an identified parent) to a plus-group at 25, when growth is assumed to stop. Length-at-age-and-sex follows a *t*_12_ distribution (heavier-tailed than a Normal to accommodate a few very large fishes) with the mean length following a sex-specific von Bertalanffy curve, and with constant CV across ages. Although fishing mortality is high on subadult SBT, it is very low on adults (much lower than natural mortality); therefore, we assume constant mortality rate *z* over age, year and sex.

This model describes numbers in the entire adult population. However, our samples are selective, coming only from the spawning grounds where SBT spend just a part of their year, during which they lose body condition and presumably do not grow in length. We assume that selectivity (that is, probability of inclusion in the catch) follows a sex-specific logistic relationship with individual length, which we interpret as average duration on the spawning grounds.

Thus far, our model is typical of an age-structured stock assessment. Our CKMR application requires two further items in order to compute equation (2), the individual proportion of total reproductive output. First is the ability to hindcast individual adult size back from year of its capture to the year of its potential offspring's birth. We do this by assuming that each individual follows a unique von Bertalanffy growth curve with an individual *L*_∞_ but sex-specific *k* and *t*_0_; note that this is consistent with the constant CV model for length-at-age. Second is a model for annual fecundity (effective reproductive output) by length and sex. For females, we multiply the average duration on spawning grounds (that is, the selectivity curve) by the estimated daily egg biomass released^22^. There is no corresponding daily output data for males, but the male parameters are statistically estimable from the overall model, assuming that male and female adult mortality rates are the same; the male fecundity-at-size parameter estimates are very uncertain, but have little impact on estimates of other parameters. This second item is specific to SBT because of the spawning-ground fishery, the general reproductive biology and the available background data; it allows the separate estimation of adult selectivity and adult mortality, which for SBT is dominated by natural mortality and would otherwise be difficult to estimate.

### Data and likelihood components

The data to be fitted are (i) length-, sex- and age-composition data from catches of adults on the spawning grounds between 2002 and 2010, (ii) the results of each pairwise POP comparison, as well as (iii) the underlying data for the daily egg output studies^22^. Unlike a typical stock assessment, we do not use any relative abundance (for example, CPUE) or total catch data.

The log-likelihood for the entire data set consists of four independent components


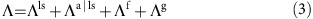


where ‘ls' is the length- and sex-frequency data (sample sizes downscaled to account for interannual overdispersion), ‘*a*|ls' is the age-frequency data (from randomized subsamples by length and sex), ‘*f* ' is the daily fecundity data^22^ and ‘*g*' is the (summarized) genotypic data. The first three are quite standard in fishery work, but the genotypic component is new. It consists of a sum of independent Bernoulli log-likelihoods over all usable comparisons between one adult *a* and one juvenile *j*, each with a binary outcome *k*_*aj*_=1 if *a* and *j* are a POP, or 0 if not:


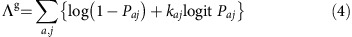



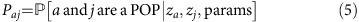


where *P*_*aj*_ is the *a priori* probability that *a* and *j* are a POP. This probability is based on the proportion of total reproductive output from *a* in the year of *j*'s birth (equation 2), taking account of length/sex/age/date of capture (and hindcasting the likely size of *a* at *j*'s birth based on *a*'s size- and age-at-capture), and of all the population dynamics parameters. By reducing each comparison to a binary outcome (POP or not), as opposed to working directly with the likelihood ratio of the specific genotypes in the pair, each computation of Λ^g^ during model-fitting is reduced to a sum over ∼50,000 categories of length, sex and age. This is ∼1,000 times faster than working with all 38,000,000 individual likelihood ratios, and sacrifices very little information; the great majority of individual likelihood ratios are unambiguous about POP status owing to the number of loci used.

### Parameter estimation

We estimated the parameters in an Empirical Bayes random-effects paradigm. Annual 8-year-old recruitment was treated as a random variable with a constant mean, whose variance is estimated in a statistically consistent way by maximizing a Laplace approximation to the marginal likelihood^47^. Maximum *a posteriori* estimates of all other parameters were then obtained by maximizing a penalized log-likelihood conditional on the point estimate of recruitment variability; parameter variances were then obtained from the penalized Hessian. All derivatives were computed by Automatic Differentiation using the software TAPENADE^48^.

We explored several variants of this model with, for example, trends allowed in recruitment, and/or separately-estimated the mortality rate for the plus-group. Perhaps because of the limited number of POPs found to date, we did not find clear supporting evidence for or against these variants; the most important summary, the time-averaged abundance estimate, was largely insensitive to model choice. The results reported in this paper do not incorporate such model uncertainty.

### Code availability

The source code for the SBT CKMR model is available from www.csiro.au/sbt-ckmr-code.

### Data availability

Data for the SBT CKMR model are available from the corresponding author on request; the Indonesian fishery components are subject to the access conditions of the Commission for the Conservation of Southern Bluefin Tuna (http://www.ccsbt.org). Pairwise kinships are included with the CKMR model code at http://www.csiro.au/sbt-ckmr-code.

## Additional information

**How to cite this article:** Bravington, M. V. *et al.* Absolute abundance of southern bluefin tuna estimated by close-kin mark-recapture. *Nat. Commun.* **7,** 13162 doi: 10.1038/ncomms13162 (2016).

## Supplementary Material

Supplementary Information Supplementary Tables 1-5, Supplementary Notes 1-3, Supplementary Methods 1 and Supplementary References

## Figures and Tables

**Figure 1:**
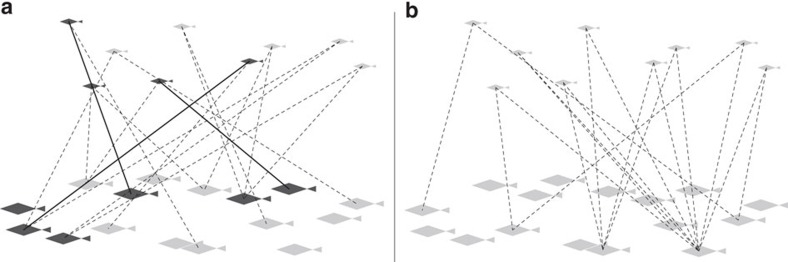
Two cartoons of parent–offspring-based close-kin mark-recapture. Reproductive variability is low in **a** but high in **b**. Each small juvenile ‘marks' its two parents among the big adults. The dark fish and lines in **a** show a sample of six adults and four juveniles, including three parent–offspring ‘recaptures'.
