# Supplementary material for: Absolute abundance of southern bluefin tuna estimated by close-kin mark-recapture
Source: Nat Commun. 2016 Nov 14;7:13162. doi: 10.1038/ncomms13162 (PMC5114523; doi:10.1038/ncomms13162)
Supplement: Supplementary Information — Supplementary Tables 1-5, Supplementary Notes 1-3, Supplementary Methods 1 and Supplementary References [file ncomms13162-s1.pdf]

Supplementary Table 1: Homozygote frequencies in the 25 primary loci used for bulk screening for POPs, as percentages.
EXP row shows expected frequencies if there were no null alleles; OBS row shows observed. The discrepancy is typically small but significant, and can be accounted for by estimating a null-allele frequency.

	3D4	B5	D10	D111	D11B	D12	D122	D201	D203	D211	D225	D235	D3	D4D6
EXP	19.8	6.8	7.1	11.8	10.7	10.8	9.7	11.7	7.5	11.4	3.4	8.5	16.8	5.5
OBS	19.8	7.3	7.3	12.2	12.3	10.9	11.4	12.4	9.0	17.0	3.7	14.8	16.8	6.7

	D541	D524	D549	D570	D592A	Z3C11A	D517	D534	D582	D569	D573
EXP	14.0	12.4	11.9	7.3	9.8	13.0	3.1	9.3	7.6	9.9	4.9
OBS	14.0	13.5	11.9	7.3	10.2	13.4	3.4	10.1	7.7	45.5	30.9

Supplementary Table 2: Breakdown of all retained adult-juvenile pairwise comparisons.
This table summarizes all adult-juvenile pairwise comparisons, except for pairs with too high a false-positive probability and pairs with the adult was caught in or before the spawning season of juvenile birth. Comparisons are tabulated by number of loci compared (rows) and number of parentage-excluding loci (columns). Leftmost column X0 contains all true POPs except for any false-negatives, plus any false-positives. Dot means zero. OBS row is observed totals; EXP0 row is expected totals if there were no true POPs (the retention criterion for comparisons was set to 0.34 expected false-positives). Columns X5-X23 are omitted for brevity, along with rows C1-10, from which no comparisons were retained.

.	X0	X1	X2	X3	X4	>	X24	X25	TOTAL
C11	.	.	.	.	1	>	.	.	84
C12	.	.	5	42	340	>	.	.	57,000
C13	.	1	16	151	887	>	.	.	143,000
C14	1	4	61	587	2876	>	.	.	652,000
C15	.	3	42	375	1962	>	.	.	923,000
C16	2	1	18	131	966	>	.	.	1,170,000
C17	2	.	8	92	655	>	.	.	1,942,000
C18	5	.	6	65	483	>	.	.	3,063,000
C19	7	.	1	33	288	>	.	.	4,158,000
C20	2	1	1	15	131	>	.	.	5,512,000
C21	14	.	1	5	62	>	.	.	7,197,000
C22	.	.	.	.	4	>	.	.	1,170,000
C23	4	.	.	.	2	>	.	.	2,966,000
C24	2	.	.	.	4	>	214	.	5,097,000
C25	6	.	1	.	1	>	1607	139	4,123,000
OBS	45	10	160	1496	8662				38,180,000
EXP0	[0.34]	11	158	1393	8457				38,180,000

Supplementary Table 3: Breakdown of all retained juvenile-juvenile comparisons.
Juveniles compared with other juveniles; note that true POPs are impossible. Finding one false-positive, at (C13, X0), is not surprising given 0.34 expected false-positives. Rows, columns, and the retention criterion to avoid false-positives, are as for Table 2.

	X0	X1	X2	X3	X4
C10	.	.	.	.	4
C11	.	.	3	50	288
C12	.	1	11	97	654
C13	1	2	43	279	1516
C14	.	1	45	327	1662
C15	.	1	19	160	920
C16	.	1	6	42	304
C17	.	.	2	31	232
C18	.	.	2	26	169
C19	.	.	6	21	144
C20	.	.	1	14	85
C21	.	.	1	.	37
C22	.	.	.	.	.
C23	.	.	.	.	6
C24	.	.	.	1	2
C25	.	.	1	.	1
OBS	1	6	140	1048	6024
EXP0	0 .34	9 .99	134 .94	1107 .76	6208 .53

Supplementary Table 4: Observed and expected numbers of near-false-positives among juvenile samples only.
Top row is observed; second row the expected value if there were no siblings among the juveniles; the lowest two rows show what increase would be expected beyond the no-siblings case if there were 1000 half-sibling pairs and 50 full-sibling pairs respectively.

	X0	X1	X2	X3	X4
OBS	1	6	140	1048	6024
EXP no sibs	0 .34	9 .99	134 .94	1107 .76	6208 .53
+1000 half	0.47	3.92	16.02	42.72	83.73
+50 full	1.29	4.93	9.23	11.23	9.97

Supplementary Table 5: Preliminary number of usable pairwise comparisons before re-scoring.
This shows raw pairwise comparison results, before manual re-scoring of genotypes in marginal cases. Rows and columsn are as for Supplementary Table 2. Columns with more than two excluding loci are omitted.

.	X0	X1	X2
C11	.	.	.
C12	.	.	5
C13	.	2	16
C14	1	4	61
C15	.	3	42
C16	1	1	18
C17	3	.	7
C18	5	.	7
C19	7	.	1
C20	2	1	1
C21	14	.	1
C22	.	.	.
C23	3	1	.
C24	2	.	.
C25	6	.	1
SUM	44		.

Supplementary Note 1 Capture probability and avoiding bias

CKMR with POPs requires computation of $\mathbb{P}[i \text{ is } j\text{'s mother/father}|z_i, z_j, \theta]$, where θ are the model parameters and(z_i, z_j) are all relevant covariate data on i and j (i.e. omitting the genotype information used to determine POP status)— including the fact that i and j were sampled. Some components of z may not be measured (e.g. adult age), and if so it is necessary to integrate over possible unobserved values; otherwise, bias can result, essentially for the same reason that "unmodelled heterogeneity of capture probability" affects Jolly-Seber-type mark-recapture[1,2]. The question to consider is: can any unmeasured covariate affect *both* the probability that an adult can be sampled, *and* the expected number of its offspring (i.e. the chance of it being tagged by virtue of an offspring being caught in the juvenile sample)? For example, if the more fecund fish in the right-hand panel of Figure 1 are also more likely to be caught, then applying the naive 2/N-based estimate would give a negatively-biased estimate of adult abundance; the naive version fails to fully condition on the covariate data, because the mere fact that an adult occurs in the sample now increases its expected number of offspring in the juvenile sample. Similar bias arises in conventional mark-recapture when some individuals are (or become) 'trap-happy'[2]. Note that the phenomenon has to occur twice, both at 'marking' and at 'recapture', to cause any bias. Since CKMR involves different animals on each occasion, the risk is somewhat mitigated compared to individual mark-recapture, but cases such as stock structure could certainly cause bias unless correctly allowed for when probabilities are computed

In the case of SBT, we assume that adult length affects residence time, which proportionally affects both selectivity and annual fecundity (the latter being also affected by daily fecundity, which is known for females). Age is assumed not to be directly related to residence time (i.e. given length, age is irrelevant). However, it is still necessary to take account of age in equation (2), because the historical length of an adult (in the year when a juvenile was born) is affected not just by its current length, but also by whether it is a fast- or slow-growing fish overall, which can be assessed by taking into account its age. Although length (and sex) is measured for all sampled adults, age is not, so for adults where age is not measured, the posterior distribution of age given length and sex must be used to integrate equation (2).

Supplementary Note 2 Precision and design

The precision of a CKMR abundance estimate (or, say, the mean of a time-series of abundance estimates) will depend somewhat on which other parameters need to be estimated and what data is available, but primarily on the number of POPs (P). For a large population, this will approximately follow a Poisson distribution so that the lower bound on $CV\left(\hat{N}_{\text{adult}}\right)$ is $1/\sqrt{\mathbb{E}[P]}$; thus, a 15% CV on abundance requires at least 50 expected POPs. In a scaled-up version of Figure 1, where $\mathbb{E}[P] = 2m_A m_J / N_{\text{adult}}$, this entails about $10\sqrt{N_{\text{adult}}}$ total samples if split optimally (equally) between adults and juveniles. For large N, this means that sampling will be "sparse" (i.e. most samples will not be part of a kin-pair, and there will be a negligible proportion of kin-triads), so that pairwise comparisons are almost statistically independent (Supplementary Note 3).

These simple formulae can be useful for study design, since $\mathbb{E}[P]$ can be simulated easily using guesstimates of demographic parameters without needing to fit any statistical models. Sample collection is often cheaper than genotyping, so a wise strategy is to collect more samples than thought necessary, and simply to stop genotyping when an adequately precise estimate has been obtained.

In longer-term studies, where the aim is time-series monitoring rather than just a one-off estimate, the annual sample size requirement would be lower, because the expected number of POPs is roughly *quadratic* in the number of years (since all pairwise comparisons are used). Eventually an equilibrium will be reached because, in pairs separated by many years, the chance of a parent having died before being sampled renders the information content

minimal; but in our SBT study, simulations indicate that the "quadratic benefit" will continue to apply for several more years.

Supplementary Note 3 Approximate independence for variance estimation

This section examines when it is reasonable to treat the set of pairwise comparisons as statistically independent for purposes of variance estimation. Formally, this means checking that the joint expected Fisher information of all pairs is approximately equal to the sum of expected Fisher information from individual pairs. Equality will never apply exactly, because each juvenile cannot have more than one mother nor one father (so, once that parent is found, then comparisons of that juvenile against other adults of the same sex are uninformative), and because some juveniles may be siblings (so, if the adult happens to be the parent of one of the juveniles, it will also be be the parent of the other; the two comparisons are clearly not independent). Nevertheless, approximate equality will hold if the population is large enough relative to the sample sizes, as shown below. A similar situation applies in conventional single-sample mark-recapture: each animal in the second sample of m_2 can match at most one animal in the first sample of m_1, so sampling is without replacement and it is not strictly correct to say that there are $m_1 m_2$ independent comparisons. However, for large populations sampled sparsely, the true hypergeometric distribution of the number of recaptures is well-approximated by a Binomial distribution, which corresponds to an assumption of independence.

For CKMR, it is easiest to examine independence for the cartoon scenario in Figure 1 but restricted to female adults only, so that the *a priori* chance of a MOP (Mother-Offspring Pair) is $1/F$ where F is the number of adults (implicitly all female). For brevity, assume equal sample size m for juveniles and adults. Clearly, if each juvenile and each adult was involved in just one comparison, all comparisons would be independent; however, each animal is involved in many comparisons, so independence is not guaranteed. It suffices to consider two scenarios, in each case computing the true and approximate joint Fisher information:

1. The same juvenile sample j^* is compared to all m adults, versus comparing a different juvenile to each adult.
2. The same adult sample a^* is compared to all m juveniles, versus comparing a different adult to each juvenile.

Let C_{aj} be the outcome of a pairwise comparison between adult a and juvenile j: 1 if MOP, or 0 otherwise. Let $C_J \triangleq \sum_a C_{aJ}$ be the total number of MOPs found comparing one particular juvenile J to all adults; clearly $C_J \in \{0,1\}$ since animals have only one mother. We have

$$\begin{aligned} \mathbb{P}\left[C_J = 0\right] &= \frac{F-1}{F} \times \frac{F-2}{F-1} \times \cdots \times \frac{F-m}{F-m+1} \\ &= \frac{F-m}{F} = 1 - \frac{m}{F} \qquad \text{(S1)} \\ \mathbb{P}\left[C_J = 1\right] &= \mathbb{E}\left[C_J\right] = m/F \qquad \text{(S2)} \end{aligned}$$

The log-likelihood Λ_J for the Bernoulli-distributed observation c_J is a function of the unknown parameter F,

given by

$$\begin{aligned}\Lambda_J(F) &= c_J \log(m/F) + (1-c_J)\log(1-m/F)\\ &= c_J \log m - c_J \log F - (1-c_J)\,mF^{-1} + (1-c_J)\,O\left(m^2F^{-2}\right) \qquad \text{(S3)}\\ \implies \frac{d\Lambda_J}{dF} &= -c_J F^{-1} + (1-c_J)\,mF^{-2} + (1-c_J)\,O\left(m^2F^{-3}\right)\\ \implies \frac{d^2\Lambda_J}{dF^2} &= c_J F^{-2} - 2(1-c_J)\,mF^{-3} + (1-c_J)\,O\left(m^2F^{-4}\right)\\ \implies \mathbb{E}\left[\frac{d^2\Lambda_J}{dF^2}\right] &= mF^{-3} - 2\left(1-mF^{-1}\right)mF^{-3} + O\left(m^2F^{-4}\right)\\ &= -mF^{-3} + O\left(m^2F^{-4}\right) \qquad \text{(S4)}\end{aligned}$$

The independence version consists of m IID comparisons each with probability-of-success F^{-1}, and it can be shown that the Fisher information is $-mF^{-3} + O\left(mF^{-4}\right)$, with the same leading term as equation (S4). The second-order term does differ, and is larger in equation (S4) by a factor m, but will be negligible in efficiently-designed surveys of large populations. To see this, recall from the Introduction that the required sample size m is something like $15\sqrt{F}$ (adjusted to female adults only). The constant of proportionality hidden by the $O(.)$ notation turns out to be 3, so the ratio mF^{-1} between the first and second terms might be around $45/\sqrt{F}$. Thus, as long as F is, say, more than 100000, the correct second term in equation (S4) is much smaller than the first, and the "independence" approximation (which uses a too-small approximation to the second term) will be adequate.

In case 2, provided that none of the juveniles are full siblings or maternally-linked half-siblings, then at most one of the comparisons can yield a MOP, and exactly the same sampling-with/without-replacement argument can be applied. If some of the juveniles share a mother, so that the m juveniles have only $m^* < m$ distinct mothers, then the Fisher information about F is only that of m^* independent (actually, sampling-without-replacement) comparisons. To compute the expected information, we need to know what m^* is likely to be. In a cartoon world, the chance that a second randomly-chosen juvenile will have the same mother as a first juvenile is $1/F$, and the chance that a third juvenile will have again the same mother is negligible, so m comparisons will yield on average about $m^* = m\left(1 - F^{-1}\right)$ distinct mothers. Unless F is small, this source of non-independence will not matter, even in the cartoon. In more realistic settings, the expected value of m^* would decrease due to reproductive variability among females, and to non-independence in juvenile samples (e.g. if siblings commonly school together at the age they are caught). The larger F is, the less those issues are likely to matter (because they only change the multiplier on F^{-1}, which is still much less than 1), but an *a priori* argument must be made on a case-by-case basis. *A posteriori* analysis of the SBT results rules out high levels of sibship among the juvenile sample.

Supplementary Methods 1 Reliability of POP identification

False-positives, error rates, and exclusion criteria

There are about 41,000,000 potential pairwise comparisons of adults to juveniles. Although the per-comparison chance of a false-positive POP (zero parentage-excluding loci) using all 25 loci would be just 2.4×10^{-13}, not all loci can be scored for all fish, so some comparisons have substantially higher chance of yielding a false-positive. We discarded those pairs with the highest *a priori* false-positive probabilities (based on which loci were scored, not on what genotypes were observed) until the expected total of false-positives was 0.34 (<1% of the total number of POPs found, and implying a <1% chance of two or more false-positives), leaving about 38,000,000 pairs to be checked for POPs. This is summarized in Supplementary Table 2, with the POPs visible in the leftmost column. In the lower rows, there is clear separation between the POPs and the morass of unrelated pairs, which also lets us

assess false-negatives arising from genotyping error. If false-negatives were common, then some of the true POPs in the lower left-hand corner would be shifted into the neighbouring column(s), which has not occurred. While the possibility of false-negatives cannot be entirely ruled out in the upper rows where no gap would be seen, detailed statistical analysis indicates an upper 95% CI of 1 false-negative (see final subsection below).

For comparison, Supplementary Table 3 shows the same process applied only to the juvenile samples, which cannot contain any true POPs; the leftmost column, which contained 45 pairs in Table 2, has just 1 pair in Supplementary Table 3, and that pair was scored at relatively few loci so its false-positive probability is higher.

The observed and predicted column totals match well in both tables, and in particular with no excess in the number of pairs with one or two parentage-excluding loci, so the calculations appear sound and there is no reason to expect that the number of POPs has been substantially underestimated due to false-negatives, nor overestimated because of false-positives from unrelated pairs and/or non-POP close-kin.

Bias in POPs is thus not a concern, but in principle overdispersion could still arise if half- or full-sibs are common among the sampled juveniles, since comparisons of one adult against members of a sib-group are not statistically independent. There are too few loci to detect half-sibling pairs directly, but a substantial incidence of full- or half-sibs would lead to excess of observed over expected in the first few column totals of Supplementary Table 3. No such excess is seen. An upper confidence interval from the analysis below (Supplementary Methods 1) shows that sibship in the juvenile samples cannot be common enough to cause serious overdispersion in the number of POPs. In other words, the situation of Fig 1 RHS does not apply.

Genotyping error rates per se are not our focus— the key point is reliable identification of POPs, and the above discussion captures the overall impact of any errors. However, the POPs themselves carry additional information on the nature of "errors". Of the 45 POPs (all of which were re-scored for validation), 9 have one locus where the two fish were scored as different homozygotes (i.e. one AA, the other BB), i.e. apparent parentage-exclusions consistent with inheriting a null allele; all the loci involved have non-zero estimated frequency of null alleles, based on apparent homozygosity rates. To cope with the possibility of null alleles, we used throughout a weakened parentage-exclusion criterion that ignored such apparent-double-homozygote mismatches (see Methods). Most of the 9 POPs involved were scored at 17 or more loci, so are most unlikely to be false-positives; thus the modified parentage-exclusion criterion appears to be genetically appropriate, at least in this study.

Non-parental close-kin

Non-POP close-kin pairs are likely to have fewer parentage-excluding loci than "unrelated" pairs (of course, no pair is *completely* unrelated) and therefore in principle might contribute additional false-positive POPs that are not accounted for in the expected-value calculations of Supplementary Table 2. However, given the large number and high allelic variability of our loci, the chances are quite low. For adult-juvenile comparisons, the next-closest plausible kin-pairing is *a priori* grandparent-grandoffspring pair (GGP) or half-sibling pair (HSP) where the birth-date is separated by at least the adult age-at-maturity. At least in a fairly short study (less than one generation), adult-juvenile GGPs and HSPs must be less common than POPs because of the many years of extra adult survival required by the grandparent or parent respectively— i.e., the same reason that just one parent-offspring pair was found among 11,000,000 adult-adult comparisons alone. Aside from scarcity, HSPs/GGPs are also intrinsically unlikely to give a false-positive POP in this study simply because of the number of loci used; on average, a GGP or HSP will coinherit a shared allele at half the loci (though this will vary between pairs), and in a typical 20-locus comparison in this study, only 0.002% of HSP/GGPs that coinherit at 10 loci would fail to exclude parentage at any of the remaining 10 by chance. A solitary adult-juvenile HSP or GGP does nevertheless seem the most likely explanation for the (C25,X2) entry; this has since been checked using an independent panel of SNP markers, confirming that the pair was clearly not a POP, and that the level of allele-sharing was consistent with GGP or HSP.

The next-closest pairing is of the (half)-uncle-niece type; these could be commoner than POPs on demographic grounds, but their degree of kinship is much weaker than for HSP/GGP, and the false-positive POP probability is negligible.

In practice, if there was an appreciable number of false-positives from non-POP close-kin, then there would also be much larger numbers of near-misses that would be apparent in the X1 and X2 columns of Supplementary Table 2.

Bounding the proportion of siblings among the juvenile sample

Supplementary Table 3 shows comparisons amongst juveniles only, looking for POPs which by definition cannot be present, and using the same criterion as for adult-juvenile comparisons in Supplementary Table 2; the point is that false-positives are indeed as low as theory predicts. But Supplementary Table 3 can also be used to bound the extent of sibship (full- and half-siblings) within the juvenile sample. If the proportion of sibs was substantial, then more pairs than expected would have a small number of parentage-excluding loci, so the first few column totals would be higher. Examples are shown in Supplementary Table 4, where it is clear that as few as 50 juvenile full-sibling pairs or 1000 juvenile half-sibling pairs would inflate the expected values particularly in the X1 column to values considerably higher than observed; e.g., with 1000 half-sibling pairs and no full-siblings, the probability of seeing as few as the 6 observed X1 pairs is 0.015.

The impact of juvenile sibship on variance estimates (by reducing the effective number of independent comparisons) depends not just on the number of pairs, but also on how the groups are arranged; for example, 1000 juvenile half-sib pairs could come from anywhere between one group of about 45 juveniles sharing one common parent, or from 2000 juveniles in 1000 pairs. The latter would obviously be the worse scenario for non-independence, since about 1/8 of all juveniles would be involved, but even then it turns out that the overall CV computed assuming independence would still only be about 12% too low (not 12 percentage points). A further bound on the extent of sibship comes from the fact that no triads were encountered among the 45 POPs found; if the number of HS-groups was as high as 800, for example, then we would expect to see 4 triads, and the probability of encountering zero would be just 0.014.

Rigorous bounds on false-negatives

Genotyping error rates, insofar as they apply to (and matter in) parentage studies, are not easy to estimate in advance; certain types of error may be heritable in the sense that, if that error is made when scoring the parent, it then it is more likely to be made when scoring an offspring of that parent. However, once a parentage study has revealed enough clear POPs— which may, as here, require large sample sizes— then a *post hoc* analysis can provide information on likely error rates and the implications for accuracy of the POP count.

We distinguish between large-scale errors, which could affect many fish at once, medium-scale errors which could affect many loci for a single fish, and small-scale errors, which can happen independently to individual loci for individual fish. Large-scale errors could arise from chimeras and mass failures of PCR on a run plate, but our protocols were able to detect and therefore eliminate these problems. Medium-scale errors could arise from contamination (easy to see, with more than two peaks at a locus) or perhaps from poor-quality DNA leading to some peaks being overlooked; however, this would likely lead to an excess of recorded homozygotes in fish with poor-quality DNA, and we found no evidence of such a relationship among fish with enough scorable loci to be included in the pairwise comparisons.

Turning to small-scale errors, the specific questions of interest are: what proportion of true POPs could have a scoring error that leads to the POP being overlooked, and what is the true number of POPs within our comparisons overall? We can estimate these quantities by comparing observed values in Supplementary Table 2 to their expected counterparts if there were no true POPs. Overall, scoring errors cannot be common, otherwise the lower part of

left-hand column of Supplementary Table 2 would smudge into the next column or two; this would be visible in the lowest rows of the Table, where the non-POPs on the right-hand side of the Table do not reach the left-hand columns. However, any smudging would not be obvious in the upper rows, where non-POPs do reach as far as the X1 column. We examine this formally in the rest of this section.

The data used for bounding false-negatives are a pre-re-scoring version of the data (Table 5), which includes some small-scale genotyping errors that were later manually rescored and corrected. The pre-rescoring data in Table 5 are very similar to Table 2, the main difference being that the C23 row starts (3,1) rather than (4,0). This is one case where a scoring error did initially cause a false-negative, but the error was detected and fixed on re-scoring. The other differences did not affect POP status of any pairs.

Likelihood for estimating false-negative rate

Let θ be the probability that a pair of fish will be a POP (so θ is inversely related to abundance, etc), and let e be the probability that one shared locus in a POP will fail the parent-offspring compatibility test[1], either through mis-scoring or mutation. Assuming scoring errors at different loci are independent[2] and equally likely[3], then the probability of f loci failing in a POP where c loci are compared, is a simple Binomial probability. Also, for a non-POP pair where c loci are being compared, let p_{cf}^{NON} be the probability that f of the loci will fail the test. For any given pair, this actually depends on the particular loci involved, and is already calculated to form the basis for the expected values in Table 2. Any given pair with c loci compared is either a POP or not, and the probability p_{cf} that the pair will fail at f loci is therefore

$$p_{cf} = \theta \left(\begin{array}{c} c \\ f \end{array} \right) e^f \left(1-e\right)^{c-f} + \left(1-\theta\right) p_{cf}^{\text{NON}} \tag{S5}$$

Therefore, if n_c denotes the number of comparisons using c loci in Supplementary Table 2, the expected value of cell (c, f) is $n_c p_{cf}$. Strictly, the distribution within each row is Multinomial, but in the first few columns the multinomial size index is enormous (millions) and p_{cf} is small, so a Poisson approximation is accurate. If y_{cf} denotes the observed number of pairs in the (c, f) entry of Table 3, then the likelihood of the first few columns up to F failures is

$$\prod_{c=11}^{25} \prod_{f=0}^{F} e^{-n_{cf} p_{cf}} \left(n_{cf} p_{cf}\right)^{y_{cf}} \tag{S6}$$

up to a multiplicative constant. The term p_{cf} involves the parameters θ and e, which can be estimated via maximum likelihood.

The bulk of the information on false-negative rates is contained in the X1 column of Supplementary Table 5 (and the X0 column, which is needed for estimating θ), with a little coming from the X2 column. In the X3 columns and beyond (omitted from the Table shown here), the noise from the increasingly large numbers of almost-false-positives swamps any signal related to false-negatives with 2, 3, etc number of failures, which will be increasingly rare.

[1]The basic test is: do they share a visible allele? We used a more relaxed version, so that AA vs BB homozygotes are also deemed (potentially) compatible.

[2]Apart from chimeras, as described and ruled out in Appendix 1, and mass failures of PCR on a run plate which would be picked up by our other QC checks, there seems no reason why independence could fail.

[3]Strictly, the probability of a scoring error that leads to rejection of POP status probably varies somewhat across loci, but there is not nearly enough data to estimate this; and since the set of loci that actually get used in a comparison is a random variable, and we are only concerned with one or two errors here, the approximation is statistically negligible.

Confidence intervals on actual false-negatives

Although the Hessian from the above likelihood could be used in the standard way to derive a confidence interval for the expected number of false-negatives in a hypothetical replicate of this study, that would be solving the wrong problem. Our interest lies in the actual number in this particular study; so, if false-negatives were very unlikely beyond the X1 column, then the number of false-negatives would be capped above by the total number of X1s actually seen, regardless of how many might be found if the study was repeated. This makes quite a difference in practice. A Bayesian argument is required to get the answer we need.

We need the probability distribution of the number of false-negatives $\#FN$ given the observed data, i.e. $\mathbb{P}[\#FN|y]$ where $y = (y_{cf} : c \in 11 \cdots 25,\ f \in 0 \cdots 1)$ is the observed numbers in the X0 and X1 and possibly X2 columns (X3 onward are irrelevant because the chances of 3 or more scoring errors is negligible). For simplicity of argument, say for now that we neglect the X2 column as well. Obviously, the maximum possible value of $\#FN$ is the observed number of X1s, in this case 12. Each of these X1 pairs is either a near-false-positive or a false-negative. The probability that an X1 pair with c loci compared is actually a false-negative rather than a near-false-positive, is

$$\frac{\mathbb{P}[\text{1 error in } c \text{ loci}] \times \mathbb{P}[\text{is POP}]}{\mathbb{P}[\text{1 error in } c \text{ loci}] \times \mathbb{P}[\text{is POP}] + \mathbb{P}[\text{match at } c-1 \text{ of } c \text{ loci}] \times \mathbb{P}[\text{is not POP}]} \tag{S7}$$

One implication is that a (C12,X1) fish is much more likely to be a near-false-positive than a (C25,X1) is, because (i) the probability of a non-POP matching by chance at 11 of 12 loci is much higher than for 24 of 25, and (ii) the chance of a scoring error is about twice as high with 25 loci as with 12.

The false-negative status of the pairs are independent given θ and e, so the total number of X1 pairs that are false-negative is the sum of (in this case) 12 independent Bernoulli (0/1) random variables, with probabilities depending on the number of loci involved. There is an algorithm due to Butler[3] for calculating the Bernoulli-sum probability distribution, which is already used in the expected-false-positive calculations. Hence, given a pair of values (θ^*, e^*), we can easily compute $\mathbb{P}[\#FN = x|y, \theta^*, e^*]$ for $x \in 0 \cdots 12$. What we actually need, though, is

$$\mathbb{P}[\#FN = x|y] = \int \mathbb{P}[\#FN = x|\theta, e, y]\, f(\theta, e|y)\, d(\theta, e) \tag{S8}$$

which can be estimated by repeatedly drawing pairs (θ^{*j}, e^{*j}) from the posterior distribution of $(\theta, e|y)$ via importance-sampling, and then averaging the $\mathbb{P}[\#FN = x|y, \theta^{*j}, e^{*j}]$ across all the draws. This requires a prior for (θ, e), which we took to be independent uniform on $\log\theta$ and logit$e$, plus of course the likelihood from equation (S6). A fully-conditioned confidence interval on $\#FN|y$ can then be found simply by inverting the cumulative distribution of $\#FN|y$.

Outcome of false-negative checks

We ran the above algorithms on the X0 & X1 columns of Supplementary Table 5. The Maximum Likelihood Estimate of the number of false-negatives was 1.95, and the 95% UCI was 2.46. As noted earlier, these estimates are *prior to* rescoring the X0, X1, and X2 (from C16 down) columns. Rescoring certainly fixed one false-negative, at (C23, X1), so the appropriate estimates and limits for the number of false-negatives in our final dataset (after re-scoring) are no more than (MLE 0.95, UCI 1.46). This is within 2% of the estimated number of POPs.

Supplementary Methods 2 QC for Consistency of Allele Size Calling

Examining the consistency of allele-size calling is fairly straightforward, and is mostly dealt with by use of an internal standard and use of an automated genotyping program developed by ABI-Life Technologies (supplier of

the DNA sequencer used for fragment separation). To further minimise inter-run variation, all size fragmentations were run on only one DNA sequencer located at the Australian Genomic Research Facility (Adelaide node). This eliminated variation occasionally observed when the same samples are run at two facilities even on the same model of sequencer.

In addition, the ABI system uses an internal size standard added to each sample from which the size curve is extrapolated for estimating allele peak length relative to the standard curve. ABI states that variation using this system ensures +/- 0.5bp accuracy from run to run. Furthermore, the GeneMapper program analyses each individual size curve for peak quality and general fit to the theoretical ideal size curve. Any discrepancies detected by the software raise flags in the analysis window and can be scrutinized in further detail. We also examined each size curve analysis as well as the individual peaks that were used to generate the size curve for each individual in a run plate to ensure another level of QC in addition to that used by the GeneMapper software.

GeneMapper uses a standard set of allele size bins used to smooth out further subtle variation and ensured easy comparison among alleles from different individuals and provided another level of QC among plates. Bin sets are developed for each locus to permit automated genotyping using the GeneMapper software. Individual bins represent a value range centred on the median length value of each allele as ascertained following sizing of an initial set of individuals. Preliminary bin sets were developed following detailed analysis of about 500 fish. These sets were designed to encompass slight variations to permit detection of gross deviations from the norm greater than +/- 1.0 bp. After genotyping about 5000 fish, the bin sets were re-assessed for consistent allele calls, and a final consensus adjustment was determined. Bins permit assignment of an integer value to the continuous-valued allele length based on the GENESCAN size standard, and permit simple comparison of allele identities among individual genotypes. A gap of one to three base pairs between bins ensures that an objective decision rule can be consistently applied to a genotype for inclusion of an allele into a designated integer bin. Alleles falling in the gap were rare and presumed to be a result of an insertion or deletion event on an individual's DNA. These were scored as "unknown genotype" but the real value could still be used for confirmation of parentage should it be required to confirm identity (not required with our samples to date).

The use of automated genotyping with a single set of GeneMapper bin-sets allowed us to detect if peaks were consistently falling outside of predetermined bins and would highlight a general problem with the running of a plate (eg. old buffer or polymer in the sequencer leading to general failure of proper electrophoresis and inconsistent separation). Runs where problems were found were re-run with new buffer and polymer; this rectified the problems in every case.

Avoidance of chimeras

Chimeric genotypes are (in this study) a composition of DNA from more than one fish, rather than (as in some other studies) DNA profiles resulting from multiple DNA in a well (two or more contaminated DNA leading to more than two alleles present for each locus). There are only two possible sources. First, a chimeric error will result from turning a run plate 180 degrees, whereby e.g. the A1 position became the H12 position. This error produces what looks like a legitimate DNA profile but made up of some loci from fish A1 mixed with the remainder of loci from H12 from the run plates that were not rotated. Second, if two run plates are swapped, the loci for those panels (but not for the other panels on the same fish) will be swapped. Clearly, these errors will lead to any POP members on the plate being overlooked, affecting 100-200 fish at a time, so it is important to catch them. Fortunately, once one is aware of these possibilities, it is fairly easy to write QC software using the check-plate results and/or the controls to detect and fix the problem. We did find both types of chimera in this study (rarely), but thanks to the QC protocols we were able to detect and fix them.

Supplementary References

1. Carothers, A. The effects of unequal catchability on Jolly-Seber estimates. *Biometrics,* 79–100 (1973).
2. Pollock, K. H., Nichols, J. D., Brownie, C. & Hines, J. E. Statistical inference for capture-recapture experiments. *Wildlife Monographs,* 3–97 (1990).
3. Butler, K. & Stephens, M. *The distribution of a sum of Binomial random variables* Technical Report 467 (Department of Statistics, Stanford University, 1993).
